# Effect of Directional Microphone Technology in Hearing Aids on Neural Correlates of Listening and Memory Effort: An Electroencephalographic Study

**DOI:** 10.1177/2331216520948410

**Published:** 2020-08-24

**Authors:** Axel H. Winneke, Michael Schulte, Matthias Vormann, Matthias Latzel

**Affiliations:** 1 Fraunhofer-Institute for Digital Media Technology IDMT, Project Group Hearing-, Speech-, and Audio Technology, Oldenburg, Germany; 2 Hörzentrum Oldenburg GmbH, Oldenburg, Germany; 3 Sonova AG, Stäfa, Switzerland

**Keywords:** electrophysiology, speech-in-noise, cognition, alpha band, spatial noise processing

## Abstract

The aim of the study was to compare the effect of different spatial noise-processing algorithms in hearing aids on listening effort and memory effort on a subjective, behavioral, and neurophysiological level using electroencephalography (EEG). Two types of directional microphone (DM) technologies for spatial noise processing were chosen: one with a wide directionality (wide DM) and another with a narrower directionality (narrow DM) to accentuate the speech source. Participants with a severe hearing loss were fitted with hearing aids and participated in two EEG experiments. In the first one, participants listened to sentences in cafeteria noise and were asked to rate the experienced listening effort. The second EEG experiment was a listening span task during which participants had to repeat sentence material and then recall the final words of the last four sentences. Subjective listening effort was lower with narrow than wide DM and EEG alpha power was reduced for the narrow DM. The results of the listening span task indicated a reduction in experienced memory effort and better memory performance. During the memory retention phase, EEG alpha level for the narrow relative to the wide DM was reduced. This effect was more pronounced during linguistically difficult sentences. This study extends previous findings, as it reveals a benefit for narrow DM in terms of cognitive performance and memory effort also on a neural level, and when speech intelligibility is almost 100%. Together, this indicates that a narrow and focused DM allows for a more efficient neurocognitive processing than a wide DM.

Speech perception in noise can be challenging particularly for individuals with impaired hearing even when fitted with hearing aids (Committee on Hearing and Bioacoustics, 1988; Klink et al., 2012; McCoy et al., 2005). Compensating for poor speech signal quality, regardless of whether due to external background noise or impaired hearing function, requires mental effort, which in turn can impair cognitive performance (Schneider & Pichora-Fuller, 2000). One purpose of hearing aids is to improve speech perception. Modern hearing aids try to separate the disturbing noise from the wanted speech signals (target). This is possible with DM systems, which attenuate all input signals that do not come from the direction of the target signal. There are different ways to implement these spatial noise processing systems, and the aim of the study is to investigate the effect of different spatial noise processing, implemented in modern hearing aids, on listening effort as well as memory effort for speech-in-noise tasks. Memory effort is conceptualized here as the amount of resources required to retain items in memory.

According to limited capacity/resource theory, the brain operates on a finite amount of (neural) resources shared by sensory, perceptual and cognitive processes (“Limited resources theory”; Kahnemann, 1973). When listening to speech-in-noise, the suboptimal signal quality has to be overcome by investing more cognitive resources, with the consequence that processing becomes more effortful (“effortfulness hypothesis”; Rabbitt, 1968). This redistribution of resources to process degraded signals leads to fewer resources being available for other concurrent and subsequent cognitive processes. In various contexts, studies have shown that more efficient sensory processing can free up resources to benefit higher cognitive processing including individuals with hearing aids (e.g., Frtusova et al., 2013; Just & Carpenter, 1992; Ng, Rudner, Lunner, Pedersen, et al., 2013; Ng, Rudner, Lunner, & Rönnberg, 2013; Schneider & Pichora-Fuller, 2000; A. Winneke, De Vos, et al., 2018; A. Winneke, Schulte et al., 2018; A. H. Winneke & Phillips, 2011).

Listening effort is a longstanding research topic (Watson, 1944) also in the context of hearing aid fitting (Pichora-Fuller & Singh, 2006). Based on the Framework for Understanding Effortful Listening (FUEL; Pichora-Fuller et al., 2016), listening effort refers to an intentional act of investing mental resources, which have corresponding neural underpinnings, in order to comprehend a (speech) signal of interest in an acoustically challenging environment. There are various approaches to quantify listening effort (McGarrigle et al., 2014; Peelle, 2018), such as dual-task paradigms (e.g., Fraser et al., 2010) and pupillometry (e.g., Wendt et al., 2017; Zekveld et al., 2010). Another promising approach is the recording of electrophysiological brain activity by means of electroencephalography (EEG). By recording and analyzing EEG data, change in effort can be quantified on a neural level and mapped onto subjective rating. The efficacy of this method has been demonstrated in previous studies (e.g., Bernarding et al., 2014; Dimitrijevic et al., 2019; Strauß et al., 2014; Strauss et al., 2010; A. Winneke, Schulte, et al., 2018; Wisniewski et al., 2017; Wöstmann et al., 2015).

The EEG signal can be decomposed into different frequency bands, which have been shown to be related to different aspects of cognitive functioning. There is evidence linking increase in cognitive effort to increased power in alpha band activity (7–13 Hz) (e.g., Klimesch et al., 2007; Obleser et al., 2012; Wisniewski et al., 2017). Results of an EEG study using an auditory Sternberg memory task (Obleser et al., 2012) showed an increase in alpha activity with an increase in signal degradation, indicating an increase in effort when listening environments are suboptimal (e.g., Klimesch et al., 2007; Obleser et al., 2012; Wöstmann et al., 2015). In a group of patients with cochlea implants Dimitrijevic et al. (2019) conducted a spoken digit in noise task with stimuli presented at an individual speech reception threshold of 50% (SRT50) while recording EEG and assessing subjective effort. The results revealed that alpha activity in the left frontal inferior gyrus was positively related to subjective effort ratings.

In studies using acoustic target signals in noise, activity in the alpha frequency band is assumed to have an inhibitory function on the interfering, irrelevant noise signal (Strauß et al., 2014). The more the noise signals interfere with the current task (i.e., understanding speech), the more the noise signal has to be suppressed in order to understand the target signal, namely, speech, better. Listening effort increases accordingly, reflected by an increase in power in the alpha band.

Interestingly, a study looking into memory capacity, also reports an increase in activity within the alpha frequency band (9–12 Hz) with increasing memory load during the retention phase of a memory task (Jensen et al., 2002). This increase in alpha power is interpreted as possibly reflecting active inhibition of further information entering areas involved in maintaining items in short-term memory (Jensen et al., 2002). Obleser et al. (2012) report a similar change in alpha power in relation to increasing memory load. In context of resource theory, this suggests that with an increase in memory load, the amount of invested processing resources to meet task demands increases as well. This investment of extra resources to overcome increasing demands or challenges could be conceptualized as cognitive or memory effort (Tyler et al., 1979). As postulated by the aforementioned limited capacity/resource theory, sensory and cognitive processes can interact (Peelle, 2018).

Numerous hearing aid approaches exist to improve speech perception. The objective of this study is to investigate hearing aid technologies that accentuate a speech source (e.g., a single speaker) in diffuse noise in terms of the effect on listening effort while listening to speech-in-noise and memory effort during the retention and retrieval phase. In this study, two different spatial noise reduction schemes were tested: the Real-Ear-Sound (RES) and the StereoZoom (SZ) DM approaches developed by Phonak. RES uses a monaural spatial noise processing technology simulating the directionality of the pinna whereas SZ works by creating a complex bidirectional network of four microphones via a wireless link between the hearing aids. SZ produces a more focused directional effect (mean directionality index = 4.7 dB) than RES (mean directionality index = −1.0 dB), reducing the interfering noise from the sides and the back (Latzel, 2013). In conversations with loud background noise (e.g., in a restaurant) a DM technology improves speech intelligibility (Picou et al., 2014). This benefit has also been shown for SZ when compared to RES (e.g. Appleton & König, 2014). A recent study with participants with mild hearing impairments has shown reduced subjective ratings of listening effort during a speech-in-noise task when using SZ compared to RES. This finding was paralleled by lower EEG alpha band activity for SZ compared to RES. Together, this indicates a reduction in listening effort for SZ and that alpha band activity might be sensitive to changes in listening effort on a neural level (A. Winneke, Schulte, et al., 2018).

Extending the findings from the previous studies, the first goal of this study was whether narrow (SZ) and wide (RES) DM technologies differ in terms of their effect on listening effort in individuals with severe hearing impairments. The previous study (A. Winneke, Schulte, et al., 2018) also revealed a decrease in subjective memory effort in narrow DM conditions. To obtain these data, participants were asked to rate their experienced effort to memorize words of the presented sentences. To follow-up on this intriguing finding, the second goal of this study was to investigate the effect of narrow vs. wide DM technology on behavioral memory performance as well as on both subjective and neural markers of memory effort during a memory retention phase.

To address these two goals, two separate experiments were conducted. The first one was designed to address listening effort and it entailed a modified version of the adaptive categorical listening effort scale (ACALES; Krüger et al., 2017). The second experiment entailed a modified version of a listening span task (LST) to measure memory performance and memory effort in a listening condition characterized by high speech intelligibility and a low level of listening effort. An EEG was recorded during both experiments.

## Hypotheses

Narrow DM technology is expected to improve speech perception and hence yield a reduction in listening effort compared to wide DM technology. This reduction will be reflected both on the subjective level (i.e., rating of experienced listening effort) as well as on the neurophysiological level. Regarding the EEG activity, we expect a reduction in power spectral density (PSD) in the alpha frequency band for the narrow compared to the wide DM as indication of a reduction in listening effort, because due to the narrow directionality less of the interfering noise has to be suppressed by the brain.

Furthermore, it is expected that memory performance will be better for narrow than for wide DM technology. As outlined earlier, previous studies have reported an increase in alpha activity in cognitively more demanding tasks (Jensen et al., 2002; Obleser et al., 2012). If improved sensory processing increases the availability of neural resources for cognitive processing such as memory retention or retrieval, it is expected that a wide DM technology leads to more cognitive or memory effort than a narrow DM and will hence be linked to an increase in EEG alpha band activity during memory processes.

## Experiment 1—Spatial Noise Processing and Listening Effort

### Materials and Methods

#### Participants

**Figure 1.**
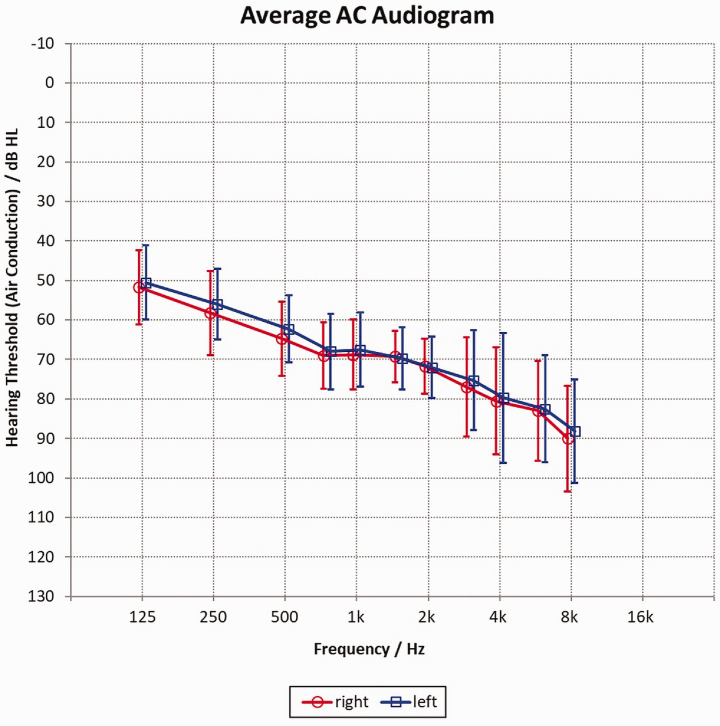
Average (N = 20) AC Audiogram for Left (Blue Squares) and Right (Red Circles) Ear Plus Standard Deviations. AC = air conduction.

A total of 20 experienced hearing aid users participated in the study (age: *M* = 65.8 years; *SD* = 14.1; 9 women). Participants had a severe hearing loss (see audiogram in Figure 1); pure tone average air conduction (PTA-AC: 0.5, 1, 2, 4 kHz; right: *M* = 71.4; *SD* = 5.3; left: *M* = 70.4; *SD* = 6.6; min avg. PTA for better ear: 61 dB). Participants were screened for normal memory functioning using the German Verbal Learning and Memory Test (VLMT) and showed age-appropriate normal memory functioning (Helmstaedter & Durwen, 1990; Helmstaedter et al., 2001). For the experiments, each participant was fitted with Naida B90-SP from Phonak, both with closed coupling with silicone-based earmolds. The default fitting rule of the manufacturer was used. Any frequency lowering was switched off. The study was approved by the local ethics committee of the Carl-von-Ossietzky University Oldenburg, Germany. Participants were compensated for participation.

#### Stimuli and Task

The speech material used in this experiment was based on the German OLSA sentence matrix test (Wagener et al., 1999). All sentences are syntactically identical and consist of five word categories (name, verb, number, adjective, and object, e.g., “Peter kauft fünf rote Blumen” [engl. “Peter buys five red flowers”]). The sentences are constructed in a random fashion based on a database that contains 10 instances for each category. Each trial consisted of a sequence of three (different) sentences (i.e., sentence triplet), after which participants were asked to rate their perceived listening effort on a scale via touch screen. The scale ranged from 1 (*effortless*) to 14 (*only noise*) based on the ACALES (Krüger et al., 2017). These values (effort scaling units—ESCU) constitute the subjective behavioral data regarding the personal experience of listening effort.

**Figure 2.**
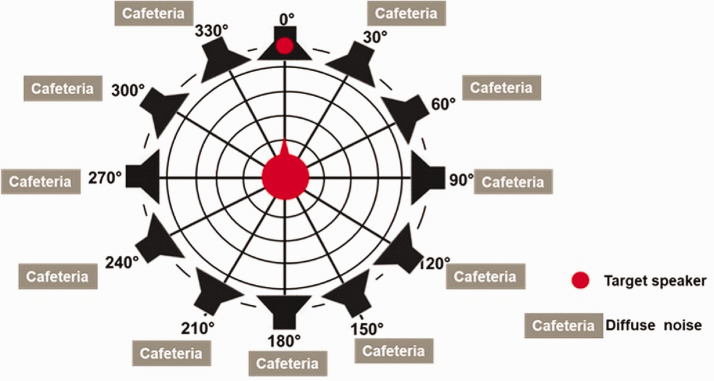
Experimental Setup.

The sentences were played in background noise at three fixed signal-to-noise ratios (SNRs). The noise signal consisted of diffuse cafeteria noise played via loudspeakers positioned at 30°, 60°, 90°, 120°, 150°, 180°, 210°, 240°, 270°, 300°, and 330° which summed up to a constant level of 67 dB(A) (see Figure 2). The noise signal was time-shifted from speaker to speaker. The loudspeakers were positioned at a distance of 1.68 m from the participant’s head. The experiment took place in a sound attenuated, dimly lit room of 26 m^2^ (4.96 m × 5.25 m).

SNR was determined by adjusting the level of the speech signal, which was presented via a loudspeaker facing the participant at 0°. The level of the speech signal was adjusted individually. First, the speech reception threshold of 50% (SRT_50_) was determined for each participant (see Procedure section). Based on this individual SRT_50_, the high SNR, medium SNR and low SNR conditions were defined as follows:

- High SNR = SRT_50_ + 10 dB.
- Medium SNR = SRT_50_ + 6.5 dB.
- Low SNR = SRT_50_ + 3 dB.

The listening effort experiment included six conditions in a 3 × 2 design with the factors SNR (low, medium, high SNR) and program (DM: narrow vs. wide).

SNR order was randomized, whereas the two hearing aid programs were presented in two blocks in a counterbalanced sequence. That is, half of the participants started with SZ and the other half with RES. Each block consisted of 30 triplets (i.e., 90 sentences) with 10 triplets for each of the three SNRs. Each sentence lasted around 3 s with an interstimulus interval of 1 s between sentences.

#### Procedure

##### First Session: Screening

After the study process was described to the participants and the informed consent was signed, an audiogram was recorded and hearing aids were fitted bilaterally based on the manufacturers’ default fitting formulas. Participants then completed the German Visual Learning and Memory Task (VLMT; Helmstaedter & Durwen, 1990) to ensure normal working memory function. This was followed by an adaptive assessment of the individual SRT_50_ using the OLSA sentence matrix task (for details see Wagener et al., 1999) at a fixed noise level of 67 dB(A) using Naida B90-SP in the RES mode. The result (=SRT_50_) is the speech level where the subject recognizes 50% of the speech.

For the LST (Experiment 2, see later), a close to perfect speech intelligibility is crucial, because otherwise it is not possible to differentiate whether performance in the memory task is due to memory processes or speech intelligibility. To determine maximal speech intelligibility, the material from the Basler sentence test (Tschopp et al., 2001) was used. The sentences consist of six to nine syllables, ending with a monosyllabic noun. The final noun is either predictable from the preceding context (high predictable, HP—e.g., “The sheep on the pasture eats grass”) or not (low predictable, LP—e.g., “What does this line mean?”). Participants listened to four lists of 15 sentences (two lists with low predictable sentences and two lists with high predictable sentences) and were asked to repeat the sentence after they heard it. The number of correctly repeated words was scored by the experimenter. The sentences were played at a level corresponding to the participant’s individual SRT_50_ plus 10 dB (high SNR) in a cafeteria background noise played at 67 dB(A). Participants were equipped with Phonak Naida B90-SP hearing aids running the RES program during this screening. Only participants who recognized 55 words or more of 60 were included in the study. This corresponds to a speech intelligibility of at least 92%.

##### Second Session: EEG Measurements

After welcoming participants to the laboratory, participants were equipped with an EEG cap. Before commencement of the experimental sessions, participants were allowed to practice the listening effort task (Experiment 1) as well as the LST (Experiment 2). When participants and the data indicated that they understood the task, the experimental sessions started. First participants completed the LST and then the listening effort task. Each task lasted about 20 min.

#### EEG Data

A continuous EEG was recorded using a 24-channel wireless Smarting EEG system (mBrainTrain, Belgrade, Serbia) while participants were performing the listening effort and LST tasks. The brain activity was recorded from 24 electrode sites mounted into a custom-made elastic EEG cap (EasyCap, Herrsching, Germany) and arranged according to the International 10–20 system (Jasper, 1958). Lab Streaming Layer (Kothe et al., 2014) and Smarting Streamer 3.1 (mBrainTrain, Belgrade, Serbia) software were used to record EEG data. The EEG was recorded at a sampling rate of 500 Hz, with a low-pass filter of 250 Hz. EEG data offline processing and analysis was conducted using EEGLab v.14 (Delorme & Makeig, 2004). EEG recordings were re-referenced off-line to a linked left and right mastoid reference. Continuous EEG data were filtered using 1 Hz to 30 Hz bandpass filter after applying a 50 Hz notch filter. Excessive ocular artifacts, such as eyeblinks, and other EEG artefacts were identified and corrected, using an independent component analysis as implemented in EEGLab.

The continuous EEG data were epoched into 2,500 ms time windows from the onset of each sentence plus a 500 ms prestimulus baseline.

To measure changes in EEG alpha activity, PSD was calculated using Welch’s (1967) method. The *pwelch* function in MATLAB (v. 2013 A, MathWorks Inc., Natick, MA) was applied for the PSD estimation. A hamming window with window length of 1 s was chosen resulting in a frequency resolution of 0.5 Hz for the PSD calculation. The PSD analysis between 3 and 25 Hz was conducted on all extracted epochs.

**Figure 3.**
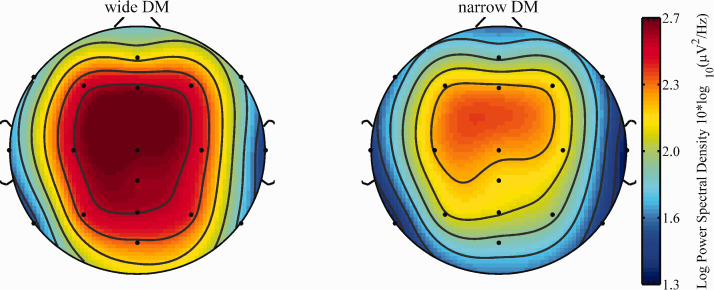
Topographical Distribution of Average Spectral Density Values (9–12 HZ) for Wide (Left) and Narrow (Right) DM Settings Averaged Across All Three SNR Conditions. DM = directional microphone.

**Figure 4.**
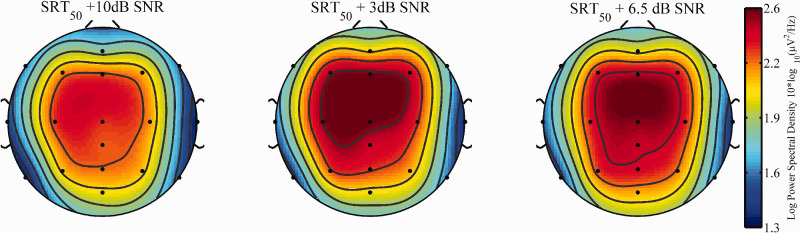
Topographical Distribution of Average Spectral Density Values (9–12 HZ) for the Three SNR Conditions SRT_50_ +10 dB (left), SRT_50_ +3 dB (Middle) and SRT_50_ +6.5 dB (Right) Averaged Across the Two Programs. SNR = signal-to-noise ratio.

Visual inspections of the topographical distribution of the activity in the alpha frequency band during sentence processing indicated highest activity around fronto-central electrodes (Figures 3 and 4) and alpha peak values between 10 and 11 Hz. Based on this observation, the average alpha PSD value between 9 and 12 Hz over the fronto-central electrode sites F3, F4, F7, F8, FZ, C3, C4, and Cz was used for the statistical analyses of the neurophysiological data.

Due to poor EEG data quality (i.e., reference mastoid electrodes were either defective or not well prepared resulting in extremely noisy data) and technical difficulties (i.e. connectivity problem with the EEG system resulting in missing data), EEG data of three participants were excluded from analysis for Experiment 1 (listening effort) resulting in a sample size of *n* = 17. For the behavioral analyses, data of all 20 participants were included.

#### Statistics

For the listening effort experiment, 2 × 3 repeated measures analyses of variance (ANOVAs; 2 [Program: narrow DM vs. wide DM] × 3 [SNR: SRT_50_ +3, +6.5, +10 dB]) were applied to the subjective listening effort ratings as well as mean PSD values for the EEG alpha frequency band averaged across fronto-central electrodes sites (F3, F4, F7, F8, FZ, C3, C4, Cz). All repeated measures ANOVAs were adjusted with the Greenhouse–Geisser non-sphericity correction (Greenhouse & Geisser, 1959) for effects with more than one degree of freedom (df) in the numerator. According to convention, uncorrected degrees of freedom, mean square error (MSE), partial eta-square (${\text{η}}_{\text{p}}^{\text{2}}$), and adjusted *p* values are reported. Significant main effects and interactions were followed by Bonferroni corrected analyses of simple effects and, unless stated otherwise, the differences reported are significant at α = .05 or below.

### Results Experiment 1: Listening Effort

With respect to listening effort in Experiment 1, the comparisons between narrow and wide DM were based on two dependent variables:

- Subjective: 14-level scale of listening effort based on the ACALES scale (Krüger et al., 2017).
- Objective: power spectral density of the EEG Alpha frequency band (9–12 Hz).

#### Listening Effort: Subjective Data

A repeated measures 2 × 3 ANOVA (2 [program: narrow DM vs. wide DM] × 3 [SNR; SRT_50_ +3, +6.5, +10 dB]) on the subjective listening effort ratings was conducted. It revealed a main effect of SNR—*F*(2, 38) = 82.10, MSE = 3.65, *p* < .01, ${\text{η}}_{\text{p}}^{\text{2}}$ = .81; a main effect of program—*F*(1, 19) = 117.47, MSE = 1.05, *p* < .001, ${\text{η}}_{\text{p}}^{\text{2}}$ = .86; and a significant interaction between the two factors—*F*(2, 38) = 4.43, MSE = .50, *p* = .03, ${\text{η}}_{\text{p}}^{\text{2}}$ = .19.

**Figure 5.**
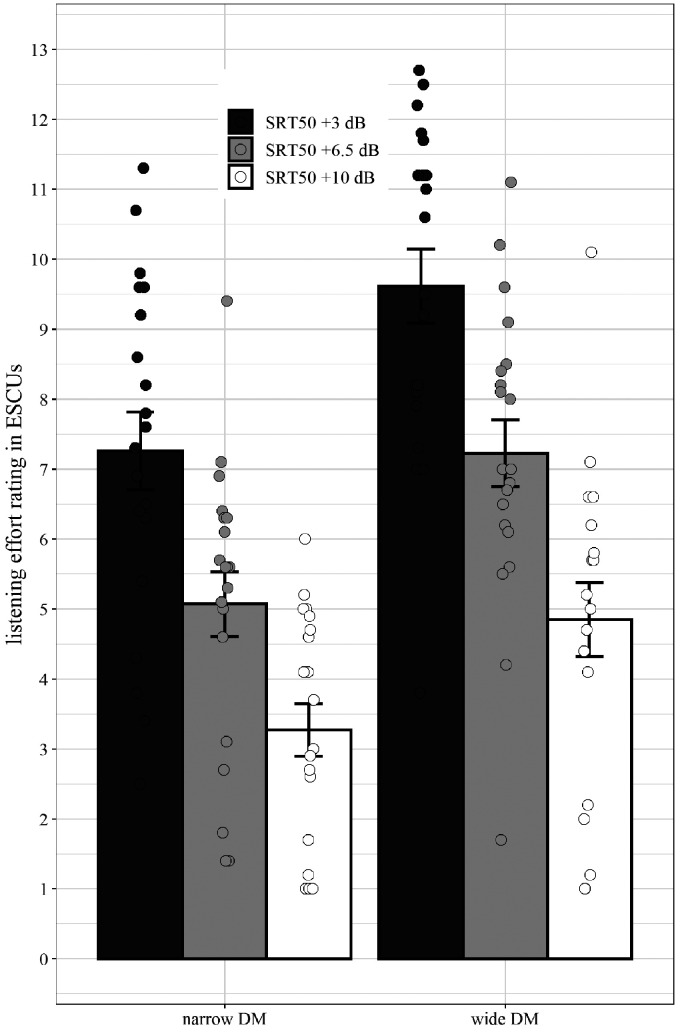
Average Subjective Listening Effort Ratings for Narrow (Left) and Wide (Right) DM for Three SNR Conditions (SRT_50_ +3dB, +6.5 dB, +10 dB). Error bars depict standard errors and circles represent individual mean values. SRT = speech reception threshold; ESCU = effort scaling units; DM = directional microphone.

The analysis showed that listening effort increases with a decrease in SNR and that the subjectively experienced listening effort is higher with the wide DM compared to narrow DM (Figure 5). The interaction is due to a stronger effect of program in the low SNR condition of SRT_50_ +3 dB SNR as compared to the high SNR SRT50 + 10 dB SNR. This can be shown by the difference values between narrow and wide DM for the low SNR condition (*m*_diff_ =2.36, *SD* = 1.06) as compared to the high SNR condition (*m*_diff_ = 1.58, *SD* = 1.18).

#### Listening Effort: EEG Data—Spectral Analysis

**Figure 6.**
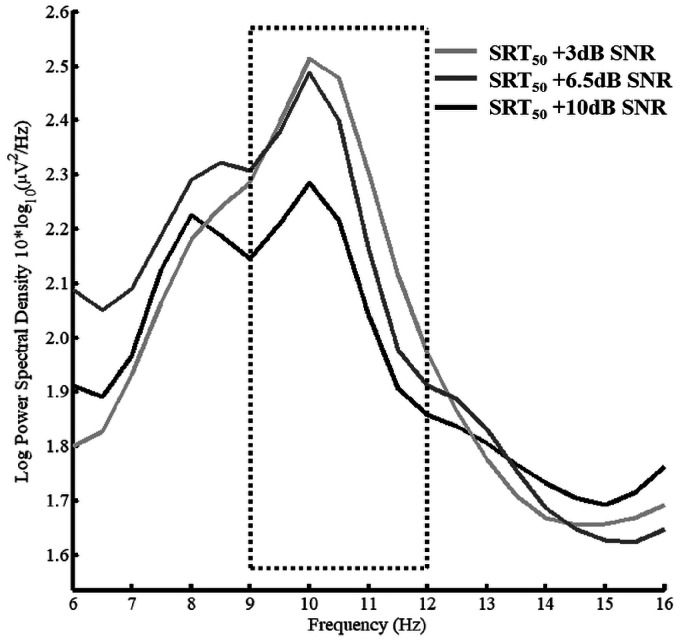
Spectral Density Curves for Three SNR Conditions SRT_50_ + 3 dB (Light Gray), SRT_50_ + 6.5 dB (Dark Gray) and SRT_50_ +10 dB (Black) Averaged Across Program and Fronto-Central Sites and Participants. Dotted box indicates 9 to 12 Hz frequency window which was used for statistical analyses. SRT = speech reception threshold; SNR = signal-to-noise ratio.

The 2 × 3 repeated measures ANOVA on the spectral analysis data revealed a trend towards a main effect of SNR—*F*(2, 32) = 2.48, MSE = .22, *p* = .17, ${\text{η}}_{\text{p}}^{\text{2}}$ = .13. This trend derives from marked differences between the low SNR condition (+3 dB) and the high SNR condition (+10 dB) as can be clearly observed in Figure 6.

**Figure 7.**
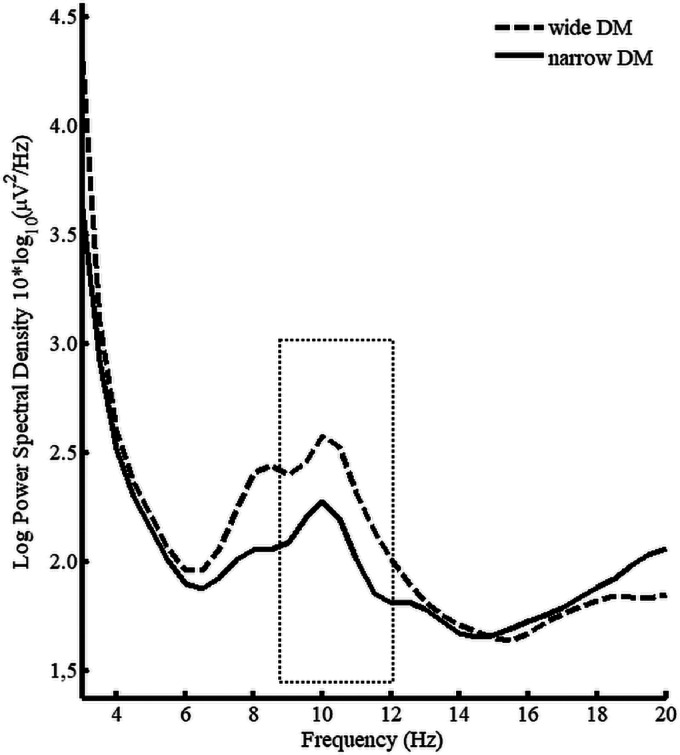
Spectral Density Curves for Wide (Dashed) and Narrow (Solid) DM Averaged Across the Three SNR Conditions, Fronto-Central Sites and Participants. Dotted box indicates 9 to 12 Hz frequency window which was used for statistical analyses. DM = directional microphone.

**Figure 8.**
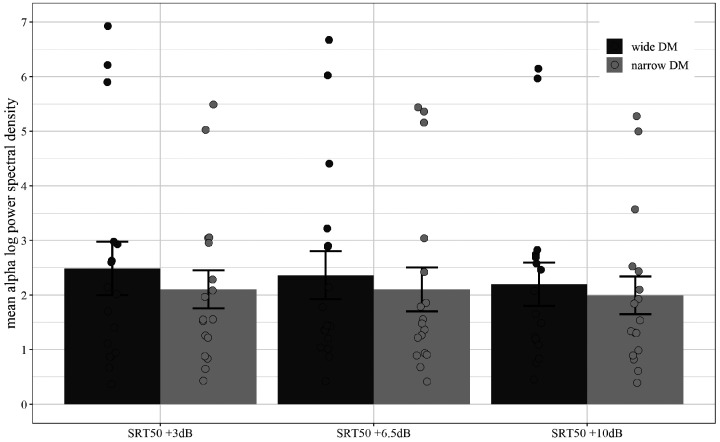
Average Alpha Spectral Density Values for Three SNR Conditions and Wide and Narrow DM. Averaged across participants, fronto-central electrodes and across frequency range 9 to 12 Hz. Error bars depict standard errors and circles represent individual mean values. DM = directional microphone; SRT = speech reception threshold.

Furthermore, the analysis revealed a main effect of program—*F*(1, 16) = 5.68, MSE = .36, *p* = .03, ${\text{η}}_{\text{p}}^{\text{2}}$ = .26—with alpha spectral density values smaller for the narrow DM as compared to the wide DM (see Figure 7). The interaction between the two factors was not significant—*F*(2, 32) = .30, MSE = .41, *p* = .63, ${\text{η}}_{\text{p}}^{\text{2}}$ = .02 (see Figure 8).

### Discussion Experiment 1

The subjective data support the hypothesis that an increase in SNR leads to a reduction in experienced listening effort. Furthermore, as predicted and in line with a previous study with participants with mild-to-moderate hearing impairment (A. Winneke, Schulte, et al., 2018), a narrow DM is associated with significantly smaller listening effort ratings compared to a wide DM. The interaction between SNR and hearing aid setting demonstrated that, the benefit of the narrow DM technology for listening effort is more pronounced in suboptimal listening environments.

The EEG results confirm the hypothesis that a narrow DM is associated with smaller alpha power than wide DM. The effect of SNR only showed a trend towards statistical significance with lower activity during the high SNR condition (SRT_50_ + 10dB) compared to the low SNR condition (SRT_50_ + 3dB). This suggests that reduction in SNR increases EEG power in alpha frequency band, which has been linked to listening effort in speech-in-noise tasks (Strauß et al., 2014; A. Winneke, Schulte, et al., 2018). Interestingly, the EEG results also show that a narrow DM can counter the effect of decreasing SNRs and reduce alpha band activity suggesting reduced listening effort. This interpretation receives further support by the subjective ratings of listening effort. At least descriptively, the EEG data suggest that the effect of the narrow DM technology is more pronounced in low SNR conditions (see Figure 8), although it should be noted that this interaction was not statistically significant and more research would be needed to solidify such a result and conclusion.

The lower alpha spectral density associated with narrow DM suggest that less irrelevant noise has to be suppressed when listening to speech-in-noise. In context of the shared resource hypothesis, this should lead to more resources available for subsequent cognitive processes such as memory function.

## Experiment 2—Spatial Noise Processing and Memory Effort

### Materials and Methods

#### Participants

The same participants as for Experiment 1 participated in Experiment 2.

#### Stimuli and Task

A German version of an LST was implemented to investigate the effect of spatial noise processing on memory effort while controlling for high speech intelligibility. The LST task is similar to the Sentence final Word Identification and Recall (SWIR) test (Ng, Rudner, Lunner, Pedersen, et al., 2013). The material in the LST task was taken from the Basler sentence test (Tschopp et al., 2001). Half of the sentences were high predictable sentences (HP) and the other half were low predictable (LP) sentences. The participants’ task was to repeat the sentence they heard and remember the last word of each sentence. After four sentences, the participants had to recall the final words of the last four sentences. Sentences were presented at the High SNR level (SRT_50_  + 10dB) to ensure a speech intelligibility level of at least 90%. The background noise and setup also corresponded to the one used in Experiment 1 (see earlier and Figure 2).

Sentence length was on average 2.4 s. The interstimulus interval between sentences was 4 s to give participants enough time to repeat the sentence. The prompt to recall the final words appeared 2 s after the final sentence was repeated and scored. The experimenter scored the number of correctly repeated final words after each sentence (words recognized) as well as the number of correctly recalled final words (words remembered). Based on this, a relative memory score was calculated. This value reflects the ratio of correctly remembered words relative to the number of actually understood final words, thereby taking speech intelligibility into account when computing memory performance. The idea behind this score is that, if a participant does not understand a word, the participant cannot remember it.

Experiment 2 included four conditions in a 2 × 2 design with the factors predictability (low, high) and program (narrow vs. wide DM). The experiment was split up into two blocks, one for each hearing aid program. The sequence of blocks was counterbalanced across participants to control for sequence effects. Each block contained 24 high predictable (HP) and 24 low predictable (LP) sentences each divided into six trials of four sentences. Trial sequence was randomized and each trial contained either high or low predictability sentences. After each block participants were asked to rate their subjectively experienced memory effort (i.e., how effortful it was to remember the items) on a scale ranging from 1 (*effortless*) to 13 (*extremely effortful*).

#### Procedure

See the Procedure section of Experiment 1 for details.

#### EEG Data

See the EEG data section of Experiment 1 for details.

The continuous EEG data were epoched into 2,500 ms time windows around the onset of each sentence with a 500 ms baseline. In addition, 2 s epochs linked to memory retention were extracted (i.e., the time window between the repetition of the final word of the fourth sentence of a trial and the prompt to recall the final words of the four previously heard sentences appeared) plus a 500 ms baseline. A power spectral density analysis between 3 and 25 Hz was conducted on all extracted epochs. The focus of analyses was placed on the EEG alpha frequency band (9–12 Hz).

**Figure 9.**
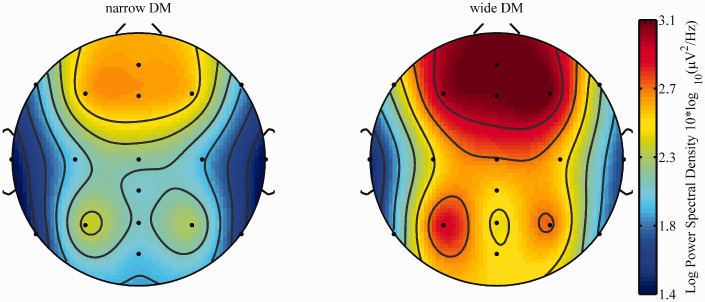
Topographical Distribution of Average Spectral Density Values (9–12 HZ) for Narrow (Left) and Wide (Right) DM Averaged Across LP and HP Sentences and Across Participants (n = 18). DM = directional microphone.

For the LST, visual inspections of the topographical distribution of the activity in the alpha frequency band during memory retention indicated highest activity around frontal electrodes (Figure 9). Based on this observation, analyses were restricted to frontal electrodes (F7, F3, Fz, F4, F8).

Due to poor EEG data quality (i.e., reference mastoid electrodes were either defective or not well prepared resulting in extremely noisy data) and technical difficulties (i.e., connectivity problem with the EEG system resulting in missing data), EEG data of two participants had to be excluded from analysis for Experiment 2 resulting in a sample size of *n* = 18. For the behavioral analyses, data of all 20 participants were included.

#### Statistics

For the LST experiment, 2 × 2 repeated measures ANOVAs (2 [Program: narrow vs. wide DM] × 2 [Predictability: high vs. low]) were applied to the power spectral density values for the EEG alpha frequency band during the memory recall phased averaged across frontal electrodes sites (F7, F3, Fz, F4, F8). The same 2 × 2 ANOVA was applied to the behavioral data (word recognition, and relative memory accuracy). All repeated measures ANOVAs were adjusted with the Greenhouse–Geisser nonsphericity correction (Greenhouse & Geisser, 1959) for effects with more than one degree of freedom (*df*) in the numerator. According to convention, uncorrected degrees of freedom, mean square error (MSE), partial eta-square (${\text{η}}_{\text{p}}^{\text{2}}$), and adjusted *p* values are reported. Significant main effects and interactions were followed by simple effect analyses.

### Results Experiment 2

With respect to memory effort in Experiment 2, the comparisons between wide and narrow DM technology were based on three dependent variables:

• Subjective: 13-level scale of memory effort.
o Objective, behavioral: percentage of correctly recognized words, percentage of correctly recalled words relative to percentage of recognized words.
• Objective, neurophysiological: power spectral density of the EEG Alpha frequency band (9–12 Hz).

#### LST: Subjective Data

**Figure 10.**
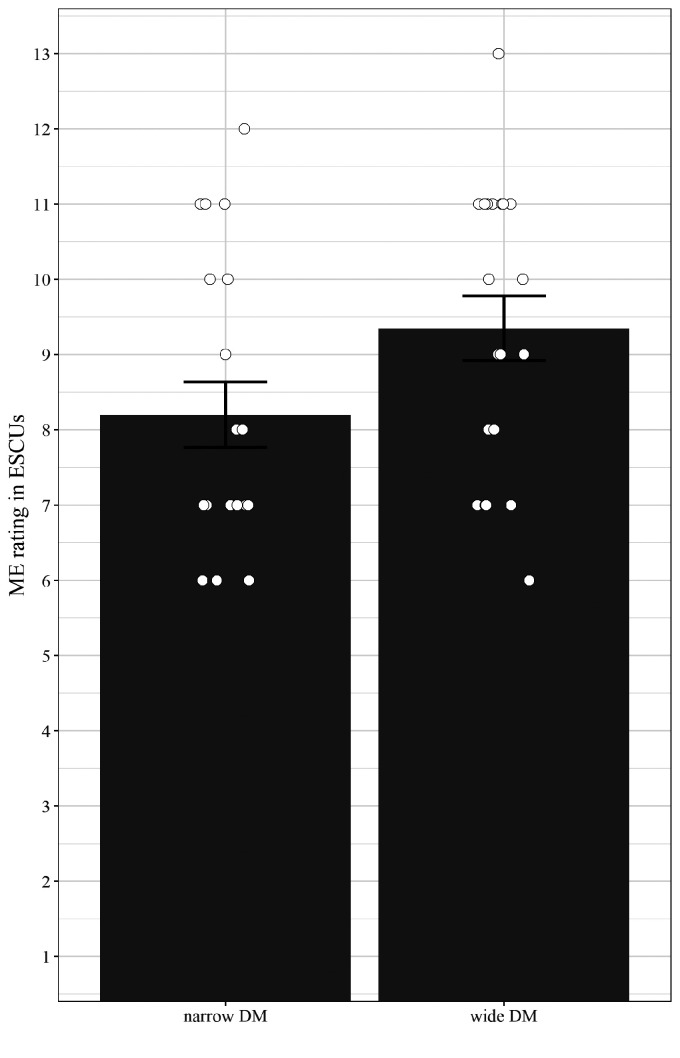
Mean Subjective Memory Effort Scores (ESCU) Averaged Across Participants and Predictability (High and Low Predictable Sentences) for Narrow (Left) and Wide (Right) Directional Microphone. Error bars depict standard errors and circles represent individual mean values. DM = directional microphone; ESCU = effort scaling units.

Simple comparisons (two-tailed paired *t* tests) regarding the subjective memory effort (i.e., “How effortful was it to remember the words?”) on a scale from 1 to 13 indicated significantly lower scores for the narrow compared to wide DM—*t*(19) = 2.93, *p* < .01; see Figure 10.

#### LST: Behavioral Data

A 2 (Program: narrow vs. wide DM) × 2 (Predictability: HP vs. LP) repeated measures ANOVA was conducted separately for the three behavioral dependent variables of (a) percentage of correctly recognized words (i.e., speech intelligibility) and (b) on the percentage of correctly remembered words relative to the percentage of correctly recognized words.

**Figure 11.**
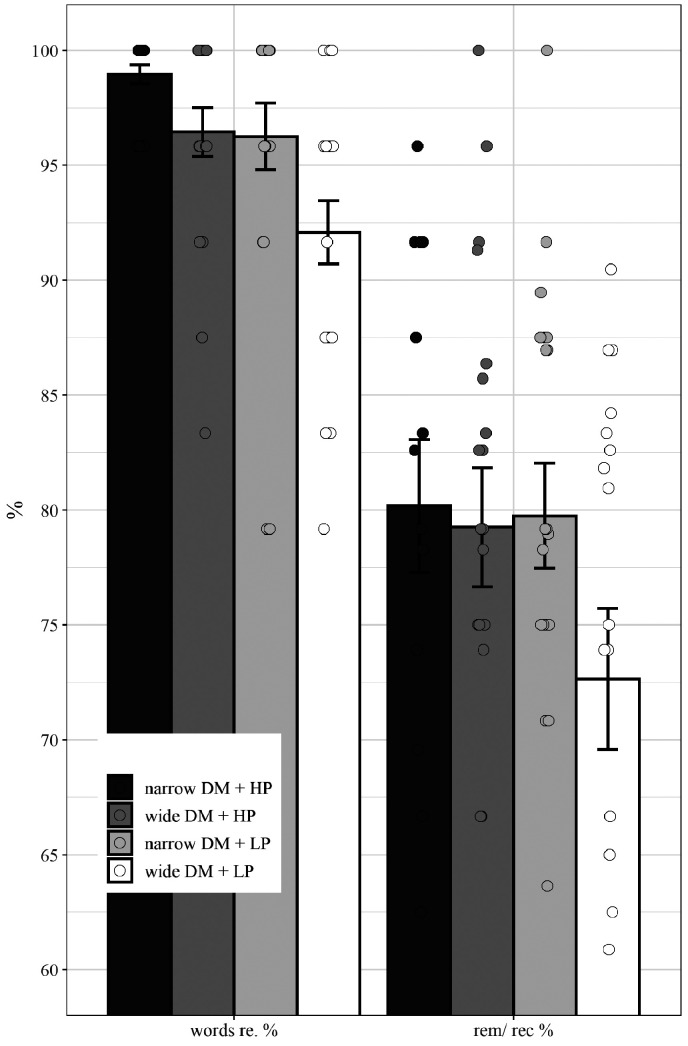
Mean Behavioral Performance Scores (Words Recognized, Remembered/Recognized Ratio) Averaged Across Participants Separated for HP and LP Sentences in Wide and Narrow Directional Microphone Setting. Error bars depict standard errors and circles represent individual mean values. DM = directional microphone; HP = high predictable; LP = low predictable.

For both measures, the analysis revealed main effects of program and predictability but no significant interaction (see Table 1). The results indicate better speech recognition and memory performance for narrow compared to wide DM and better recognition and memory performance for HP than LP (see Figure 11).

**Table 1.** Statistics Regarding Behavioral Data of LST Task.

Dependent variable	Main effect of program	Main effect of predictability	Interaction of program and predictability
Percentage of words recognized	*F*(1, 19) = 15.02, MSE = 14.80, ***p* = .001**, ${\text{η}}_{\text{p}}^{\text{2}}$ = .44	*F*(1, 19) = 14.05, MSE = 17.86, ***p* = .001**, ${\text{η}}_{\text{p}}^{\text{2}}$ = .43	*F*(1, 19) = 1.15, MSE = 12.06, *p* = .30, ${\text{η}}_{\text{p}}^{\text{2}}$ = .06
Remembered/recognized ratio	*F*(1, 19) = 7.17, MSE = 44.94, ***p* = .02**, ${\text{η}}_{\text{p}}^{\text{2}}$ = .27	*F*(1, 19) = 5.47, MSE = 45.18, ***p* = .03**, ${\text{η}}_{\text{p}}^{\text{2}}$ = .22	*F*(1, 19) = 2.42, MSE = 78.75, p = .14, ${\text{η}}_{\text{p}}^{\text{2}}$ = .11

*Note*. MSE = mean square error.

#### LST: EEG Data

**Figure 12.**
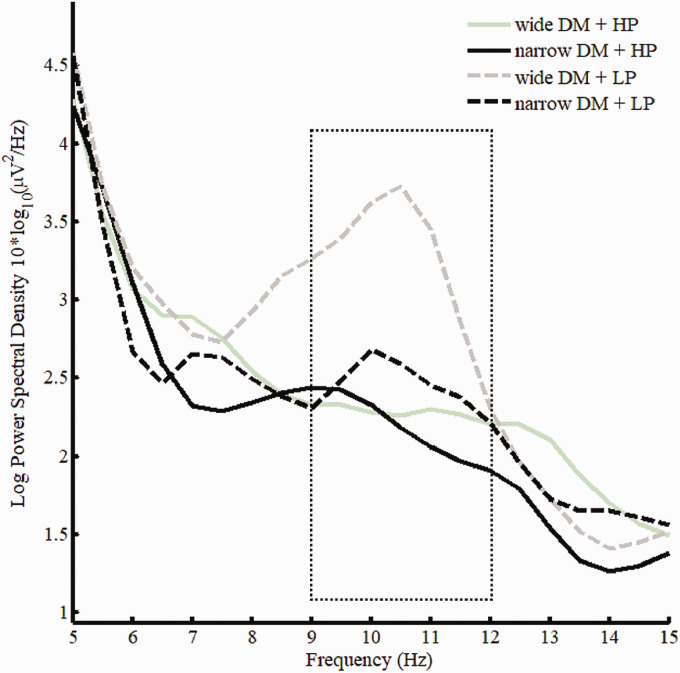
Spectral Density Curves for All Four Conditions of the LST Task Averaged Across Participants During the Retention Phase: Wide DM and HP Sentences (Solid Gray Lines), Wide DM and LP Sentences (Dashed Gray Lines), Narrow DM and HP (Solid Black Lines), and Narrow DM and LP (Dashed Black Lines). Dotted box indicates 9 to 12 Hz frequency window which was used for statistical analyses. DM = directional microphone; HP = high predictable; LP = low predictable.

**Figure 13.**
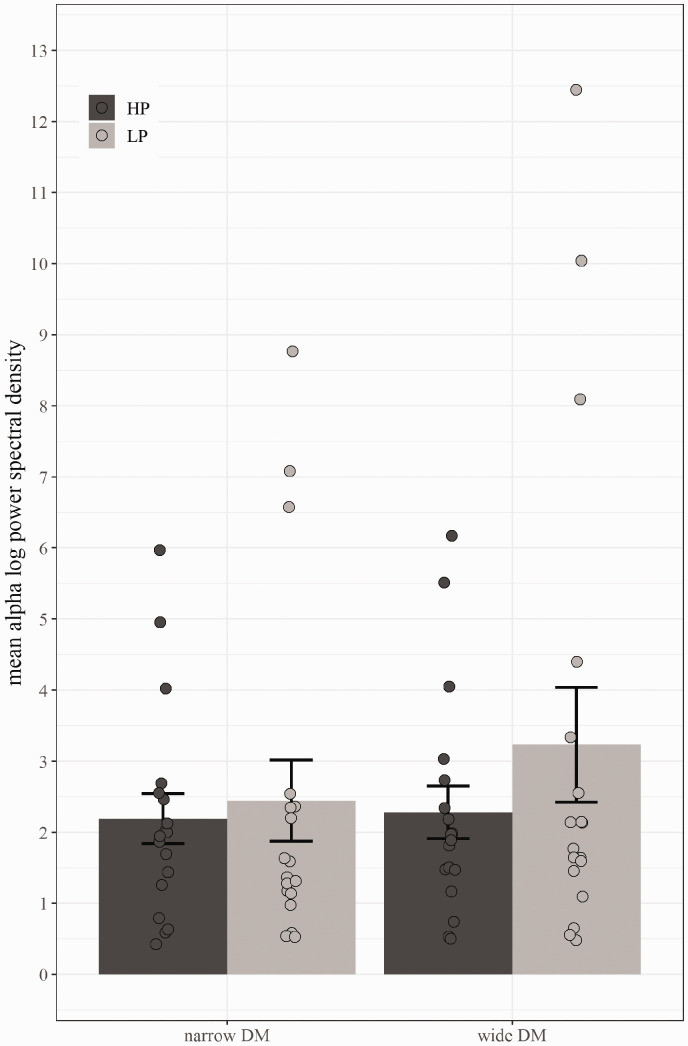
Average Alpha Spectral Density Values During the Retention Phase for Narrow (Left) and Wide (Right) DM Separated by HP and LP Sentences. Averaged across participants and across frequency range 9 to 12 Hz. Error bars depict standard errors and circles represent individual mean values. DM = directional microphone; HP = high predictable; LP = low predictable.

**Figure 14.**
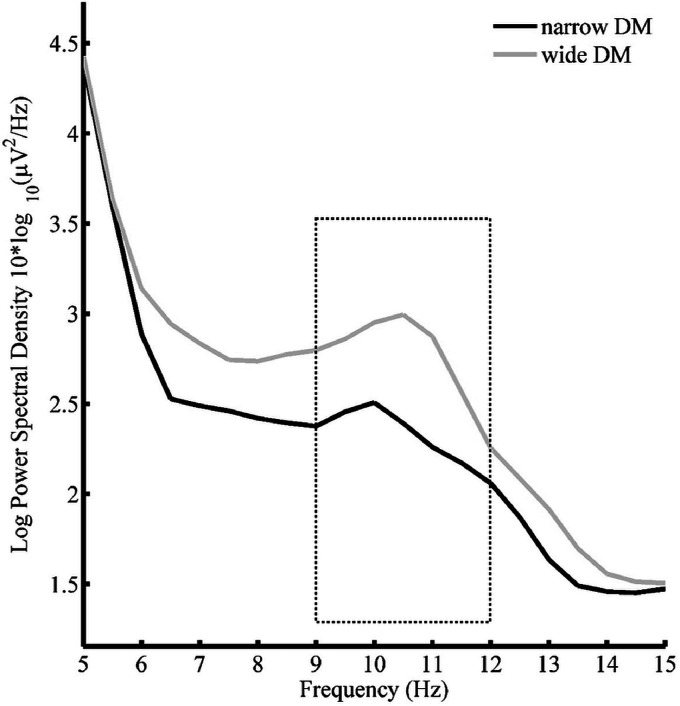
Spectral Density Curves During the Retention Phase for Wide (Gray) and Narrow (Black) DM Averaged Across High and Low Predictable Sentences, Frontal Sites and Participants. Dotted box indicates 9 to 12 Hz frequency window which was used for statistical analyses. DM = directional microphone.

The 2 (Program: narrow vs. wide) × 2 (Predictability: LP vs. HP) repeated measures ANOVA on alpha spectral density values (9–12 Hz) averaged across frontal electrodes (F3, F4, Fz, F7, F8), revealed a main effect of program—*F*(1, 17) = 5.77, MSE = .60, *p* = .03, ${\text{η}}_{\text{p}}^{\text{2}}$ = 0.25. In addition to the main effect of program, a significant Program × Predictability interaction was revealed—*F*(1, 17) = 5.28, MSE = .41, *p* = .03, ${\text{η}}_{\text{p}}^{\text{2}}$ = 0.24. The interaction is due to a pronounced effect of low predictability for the wide DM technology (see Figures 12 and 13). Figure 14 visualizes the main effect of program with more pronounced alpha activation for wide than narrow DM during memory retention collapsed across HP and LP. The effect of predictability was not statistically significant—*F*(1, 17) = 2.22, MSE = 2.94, *p* = .15, ${\text{η}}_{\text{p}}^{\text{2}}$ = 0.12.

**Figure 15.**
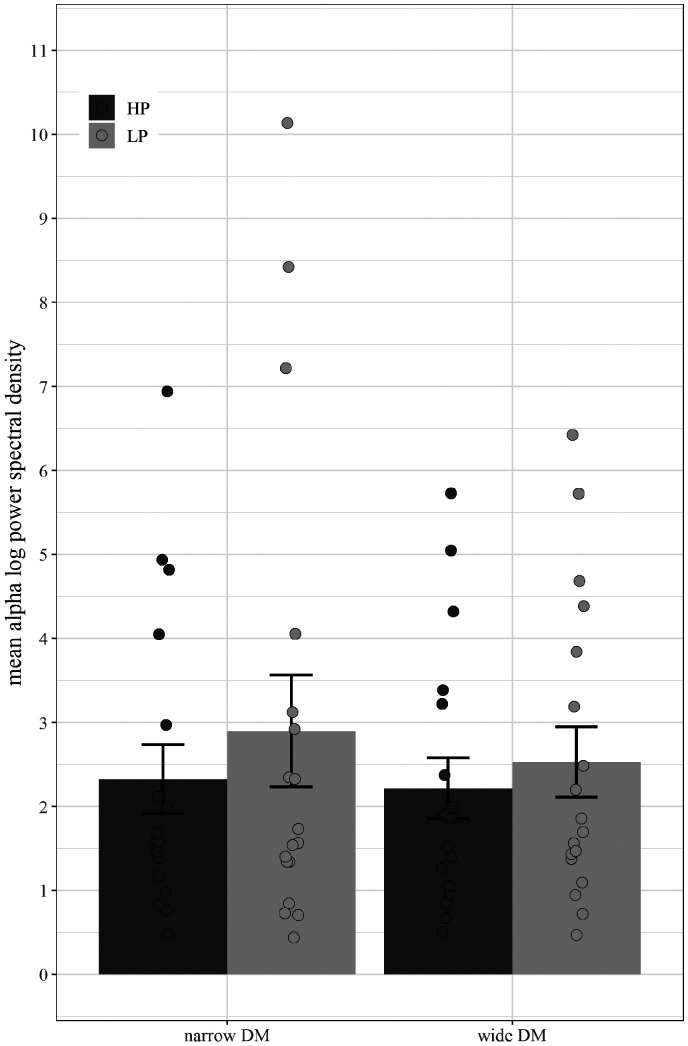
Average Alpha Spectral Density Values for Narrow (Left) and Wide (Right) DM Separated by HP and LP Sentences While Listening to Sentences of the LST at High SNR (SRT_50_ +10dB). Averaged across participants, frontal electrodes and frequency range 9 to 12 Hz. Error bars depict standard errors and circles represent individual mean values. DM = directional microphone; HP = high predictable; LP = low predictable.

To investigate whether the differences during the retention phase are simply due to listening effort while listening to the sentences at the High SNR (SRT_50_  + 10dB), we analyzed the power spectral density values in the alpha frequency band during sentence presentation (i.e., while participants were listening to the sentences). The results (see Figure 15) revealed no main effect of program—*F*(1, 17) = .84, MSE = 1.22, *p* = .37, ${\text{η}}_{\text{p}}^{\text{2}}$ = .05—and an almost significant main effect of predictability—*F*(1, 17) = 4.12, MSE = .85, *p* = .06, ${\text{η}}_{\text{p}}^{\text{2}}$ = .20. No interaction was found—*F*(1, 17) = 1.46, MSE = .21, *p* = .24, ${\text{η}}_{\text{p}}^{\text{2}}$ = .08.

### Discussion Experiment 2

The results regarding subjective memory effort confirm the hypothesis that the memory task is experienced as less effortful with a narrow DM relative to a wide DM. The behavioral accuracy data show that cognitive performance benefits from a narrow DM as memory scores were significantly better compared to the wide DM. Even though speech intelligibility was overall very high, the narrow DM outperformed the wide DM in terms of percentage of correctly understood words. In studies using the similar SWIR test, it was also shown that memory performance in noisy environments could benefit from noise reduction algorithms (Ng, Rudner, Lunner, Pedersen, et al., 2013; Ng, Rudner, Lunner, & Rönnberg, 2013).

With respect to the EEG data, the results confirm the hypothesis of reduced alpha activity during narrow versus wide spatial noise processing indicative of a reduction in memory effort at the neurophysiological level. Although Figure 13 suggests a main effect of predictability, this effect was not statistically significant. One explanation for the lack of a statistically significant effect of predictability is likely due to the fact of a larger standard error for predictability as compared to program. However, interestingly the interaction between both factors is significant because the benefit of the narrow DM is more pronounced in more difficult LP sentences (see Figure 13). This effect on the neurophysiological level, paralleled by the behavioral benefits, suggests an increase in neural efficiency when using a narrow DM technology, because fewer neural resources are recruited to achieve better cognitive performance scores.

Importantly, the results indicate that there was no difference in the alpha power spectral density between wide and narrow DM while listening to the sentences. This corresponds to the lack of differences between narrow and wide DM in Experiment 1 in the high SNR condition (SRT_50_  + 10dB). This suggests that the differences in the alpha band observed during the memory recall phase are likely not due to carry-over effects from the preceding phase of listening to sentences.

## General Discussion

The aim of the study was to investigate whether the directionality of DMs implemented in modern hearing aids reduces listening and memory effort in noisy environments. Two DM settings were selected for this study: a wide and a narrower DM. The experimental design did not include a hearing aid setting without any noise suppression as a no-support baseline. Such a condition would be an unrealistic everyday listening scenario for hearing aid users, because modern hearing aids always entail some form of noise processing. The chosen algorithm with a wide directionality (Real-Ear-Sound) is characterized by a lower degree of directivity or wider beam and is therefore a suitable reference condition to evaluate the benefits of a more directional hearing aid setting (StereoZoom) with a narrower directionality for perceptual and cognitive functioning and their neurophysiological correlates.

The target group in this study was experienced hearing aid users with severe hearing impairments. To analyze the effect of DM technology on cognition, the focus was placed on the retention/retrieval phase during a memory task (Experiment 2). That is the phase during which to be remembered items have to be stored in a short-term memory buffer just before a prompt is given to recall items from short-term memory. Given that more challenging conditions place more demand on cognitive processes, they increase cognitive effort (Obleser et al., 2012; Tyler et al., 1979), conceptualized here as memory effort.

The two concepts of interest, namely, listening effort as well as memory effort, were addressed in two separate experiments. In addition to subjective and behavioral performance scores, neurophysiological parameters linked to the two concepts were analyzed by using EEG.

The results of the listening effort experiment showed an increase in subjective ratings of listening effort with decrease in SNR as well as a clear benefit (i.e., lower scores) for the narrow DM over the wide DM. Based on previous studies (e.g., Obleser et al., 2012; Strauß et al., 2014; A. Winneke, De Vos, et al., 2018; Wisniewski et al., 2017), changes in power spectral density in the EEG alpha frequency band was chosen as electrophysiological marker associated with listening effort. There was no statistically significant linear increase in alpha power with decrease in SNR but the trend clearly showed that the lowest SNR (SRT_50_  + 3dB) was associated with stronger alpha activity than the highest SNR (SRT_50_  + 10dB). This is in accordance with the hypothesis and previously reported results and supports the notion that activity in the alpha band is associated with listening effort in speech-in-noise tasks.

For example, Wöstmann et al. (2015) manipulated acoustic degradation of target stimuli and found that alpha power decreased with increasing acoustic detail which is in line with the results for SNR as well as the effect of program in Experiment 1. As outlined in the article by Strauß et al. (2014), the functional role of the alpha band is to inhibit or suppress irrelevant background noise (Strauß et al., 2014; A. Winneke, De Vos, et al., 2018; A. Winneke, Schulte, et al., 2018). Consequently, alpha power should be larger in the low compared to the high SNR conditions. The same argument could explain the main effect of program reported in Experiment 1, which showed a significantly larger level of alpha activity when listening to speech-in-noise using a wide DM as compared to a narrow DM. In other words, the more focused, narrow directed microphone beam leads to a stronger suppression of irrelevant information before entering the auditory system. Accordingly, less distracting noise has to be inhibited by the brain. This could possibly be an explanation for lower subjective listening effort as well as the lower level of alpha power, as an objective, neurophysiological index of listening effort.

This maps onto the subjective results, which indicate an increase in listening effort when using the wide DM compared to the narrower DM setting. Both subjective and EEG results are in line with a previous study, which showed a reduction in listening effort with the narrow relative to the wide DM in participants with mild-to-moderate hearing impairments (A. Winneke, Schulte, et al., 2018). Interestingly, in this study, alpha activity is more pronounced at frontal electrode sites and not at parietal sites where the effect is often observed. Possibly this shift to frontal areas is due to the level of hearing impairment and that severe hearing difficulties and/or signal degradation require more or additional cognitive resources for speech-in-noise tasks (Obleser & Weisz, 2012; Rosemann & Thiel, 2019). Even though objective and subjective results are complementary, it should be noted that they do not necessarily measure the same underlying processes. Given that listening effort is a complex, multifactorial concept, Lemke and Besser (2016) argue that subjective and objective measures can assess different aspects of this concept.

Important to note is also that the observed effect of DM is not simply due to a change in SNR. The effect of the DM is frequency dependent (e.g., Ricketts, 2001), as the DM works better at lower frequencies. It also depends on the direction of the noise as noise from the back is reduced more compared to for example the noise from the sides (e.g., Husstedt et al., 2018). That is, the DM results not only in a spatial but also in a frequency dependent SNR improvement and is therefore rather different or more than simply changing the level of the noise.

Previously, we reported a reduction in subjective memory effort when performing a memory task in noise when using a narrow DM technology (A. Winneke, Schulte, et al., 2018). Therefore, the aim of the second experiment was to explore this finding further by including EEG to assess the neurophysiological correlates of this effect. Based on previous results, we focused on activity in the alpha band of 9 to 12 Hz during the retrieval/retention phase (Jensen et al., 2002; Wisniewski et al., 2017). To control for speech intelligibility as confounding factor in memory performance, the speech material was presented at a high SNR (SRT_50_  + 10dB). Results confirmed that intelligibility was nearly perfect.

As hypothesized, the behavioral data indicated lower subjective memory effort and better memory performance for narrow versus wide DM. In addition, EEG analysis of the memory retrieval/retention phase showed higher alpha activity for the wide as compared to the narrow DM setting. This finding is in line with findings reported by Jensen et al. (2002). They report an increase in EEG activity in the 9 to 12 Hz alpha-band during the retention phase in a memory task with increasing memory load. Interestingly, this study revealed that the increase in alpha activity was particularly pronounced for difficult sentences with low predictability of the final word compared to high predictive sentences. Similarly, Wöstmann et al. (2015) report a decrease in alpha power with increasing predictiveness of the upcoming stimulus. This effect of predictability in this study was diminished in the condition with narrow DM technology. In other words, a narrow DM can be especially beneficial in linguistically difficult listening situations. One proposed functional interpretation is that the increase in alpha power reflects active inhibition of further information entering areas involved in maintain items in short-term or working memory (Jensen et al., 2002).

The initial hypotheses were confirmed: on the one hand, the data suggest that a narrow DM benefits users by reducing listening effort, and on the other hand, it benefits cognition as seen in better memory performance and reduced memory effort both on a subjective as well as an objective level.

The results are intriguing as they show that the benefit of hearing aid algorithms go beyond improving speech intelligibility but also affect neural correlates of cognitive processing and thereby potentially enable a more efficient use of available (neural) resources. When looking at the behavioral memory performance and EEG results together, the findings suggest that listening to speech-in-noise using a narrow DM allows for a more efficient neurocognitive processing because better performance is accompanied by lower alpha power values during the retention phase. This could suggest that over a prolonged period of listening to speech-in-noise the level of fatigue could consequently be lower when using narrower DM technology. Hornsby (2013) showed an increase of reaction times in a dual task paradigm over time and interpreted this as an increase of hearing related fatigue. Future studies are needed to investigate the neurophysiological link between reduction in (listening) effort and fatigue and possibly long-term effects for health and well-being.

### Limitations

No exact speech intelligibility values for the low and mid SNR conditions were obtained, but we know from preparatory pilot studies that speech intelligibility scores were 80% and 86%, respectively. That being said, it cannot be completely ruled out that speech intelligibility factored into listening effort ratings in those two conditions. As for the high SNR conditions, it is fair to say that speech intelligibility was at or very close to ceiling. Yet, even within the high SNR, significant differences regarding subjective listening effort were obtained, indicating that differences are not merely a reflection of speech intelligibility. According to Krüger et al. (2017), listening effort ratings are influenced by speech intelligibility more at low than at high SNRs. Another factor that influences effort is motivation (Peelle, 2018). It cannot be ruled out that some participants were more motivated than others, but based on our experience of testing hearing impaired individuals and based on the observations during the experimental sessions it is unlikely that participants were not motivated to complete the experiments as instructed. Also, it should be noted that the number of trials in Experiment 2 is on the low side. This can affect the overall signal to noise ratio in the EEG signal. However, in order to not to overburden the participants and thereby introducing confounding factors such as fatigue, for example, it was necessary to compromise.

DM technology clearly indicates improvements in speech intelligibility, listening and memory effort, as well as cognitive performance. Yet, one might argue that DMs or spatial noise processing is only adequate for specific situations; that is, where there is only one sound source of interest. The downside of suppressing spatial information stemming from locations other than the source of interest can cause other acoustic information not to be perceived as well by the listener anymore and perhaps not reach the listener’s awareness, even though it could be of relevance. However, when having a conversation in a noisy restaurant, a DM enhances the signal of interest and can improve the overall quality of the conversation.

## Data Availability

Data will be made available upon reasonable request.
